# Comparative transcriptome and metabolome analysis reveal glutathione metabolic network and functional genes underlying blue and red-light mediation in maize seedling leaf

**DOI:** 10.1186/s12870-021-03376-w

**Published:** 2021-12-14

**Authors:** Tiedong Liu, Xiwen Zhang

**Affiliations:** 1 College of Agriculture, Fujian Agriculture and Forestry University, Fuzhou, 350002 Fujian China; 2 Key Laboratory of Ministry of Education for Genetics, Breeding and Multiple Utilization of Crops, College of Agriculture, Fujian Agriculture and Forestry University, Fuzhou, 350002 Fujian China

**Keywords:** Blue light, Red light, Glutathione, Transcriptome, Metabolome, *Zea mays*

## Abstract

**Background:**

Light quality severely affects biosynthesis and metabolism-associated process of glutathione. However, the role of specific light is still unclear on the glutathione metabolism. In this article, comparatively transcriptome and metabolome methods are used to fully understand the blue and red-light conditions working on the glutathione metabolism in maize seedling leaf.

**Results:**

There are 20 differently expressed genes and 4 differently expressed metabolites in KEGG pathway of glutathione metabolism. Among them, 12 genes belong to the glutathione S-transferase family, 3 genes belong to the ascorbate peroxidase gene family and 2 genes belong to the ribonucleoside-diphosphate reductase gene family. Three genes, *G6PD*, *SPDS1*, and *GPX1* belong to the gene family of glucose 6-phosphate dehydrogenase, spermidine synthase, and glutathione peroxidase, respectively. Four differently expressed metabolites are identified. Three of them, Glutathione disulfide, Glutathione, and l-γ-Glutamyl-L-amino acid are decreased while L-Glutamate is increased. In addition, Through PPI analysis, two annotated genes *gst16* and *DAAT*, and 3 unidentified genes *100381533*, *pco105094* and *umc2770*, identified as *RPP13-like3*, *BCAT-like1*and *GMPS*, were obtained. By the analysis of protein sequence and PPI network, we predict that *pco105094* and *umc2770* were involved in the GSSG-GSH and AsA-GSH cycle in the network of glutathione metabolism.

**Conclusions:**

Compared to red light, blue light remarkably changed the transcription signal transduction and metabolism of glutathione metabolism. Differently expressed genes and metabolic mapped to the glutathione metabolism signaling pathways. In total, we obtained three unidentified genes, and two of them were predicted in current glutathione metabolism network. This result will contribute to the research of glutathione metabolism of maize.

**Supplementary Information:**

The online version contains supplementary material available at 10.1186/s12870-021-03376-w.

## Background

Glutathione (GSH) is a tripeptide of cysteine, glutamic acid, and glycine, which exists in most plant tissue. Glutathione molecule is characterized by active sulfhydryl group, which is the most important functional group used as an antioxidant, radical scavenger and antidote. As a substrate for glutathione S-transferase (GST), this agent reacts with many of harmful chemical such as halides, epoxides and free radicals to form harmless inactive products [1]. Glutathione protects cell, preserves enzymes activities and proteins functions, prevents cytoplasmic and outer membranes damage [2]. These reactions prevent oxidative damage through the reduction of peroxides. Glutathione is also important as a cofactor for the enzyme glutathione peroxidase (GPX), Glyceraldehyde-3-phosphate dehydrogenase, glyoxalase, and triose dehydrogenase [3]. Glutathione participates in carbohydrate metabolism, and can activate many enzymes, such as 6-phosphogluconate dehydrogenase, thus promoting the metabolism of carbohydrate, and protein [4]. Glutathione also participates in the formation and maintenance of disulfide bonds in proteins and the transport of amino acids across cell membranes [5].

There are many ways to balance GSH metabolism. GSH may rapidly oxidize to glutathione disulfide (GSSG) under oxidative stress, but under normal conditions, it is reduced back to GSH by glutathione reductase (GR) via an reductant nicotinamide ademine dinucleotidephosphate (NADPH) -dependent mechanism both in the cytosol and mitochondria to maintain the GSH/GSSG balance [6]. The GSH-GSSG cycle shapes glutathione activity. Glucose 6-phosphate dehydrogenase (G6PD) involved in this cycle by catalyzing glucose-6-phosphate and dependent isocitrate dehydrogenase (NADP+) to 6-phospho-δ-glucono-1,5-lactone and NADPH [7]. In order to maintain low levels of reactive oxygen species (ROS), ascorbate peroxidase (APX) and GPX convert hydrogen peroxide to water in the ascorbate (AsA)-glutathione (GSH) cycle and oxidized glutathione (GSSG)-GSH cycle, respectively [8, 9]. The biosynthesis of trypanothione demands both GSH and spermidine (Spe), GSH and Spe has been shown to be growth-phase dependent even being conjugated. Therefore, spermidine synthase (SpeE) takes part in the AsA-GSH cycle as a catalyst [10]. With the participation of various enzymes, the metabolic pathway of glutathione becomes complicated when it involves in environmental stress.

Light is very important for glutathione metabolism. Light intensity and spectrum affect metabolism of glutathione and amino acids at transcriptional level [11]. Ultraviolet-C (UV-C) exposure significantly induced most of the GR activities. Contents of reduced glutathione were also augmented in response to such UV-C radiation exposure [12]. Glutathione contents and GR activities were improved by long-term red and blue continuous light, and AsA-GSH cycle did not contribute much to ascorbate accumulation under continuous light [13]. Overexpression of GR leads to increases in antioxidant capacity and resistance to photoinhibition [14]. The ratio of red/far-red light is an important regulators of GSH [15]. light-emitting diode (LED) maintain high level of glutathione in banana antioxidant system [16]. Blue and red-light are the main light sources of photosynthesis, which affect glutathione metabolic, but there is still lack of effective information. Because of the comprehensive role of light in plant growth and development, we combine transcriptome with metabolome analysis methods. By the comprehensive analyses of differently expressed genes and differently expressed metabolites of maize seedling under blue light and red light, this paper attempts to expand the signal network, discover new potential functional genes, and finally improve glutathione metabolic network.

## Results

### Enzyme activity in glutathione metabolism response to blue and red-light

**Fig. 1:**
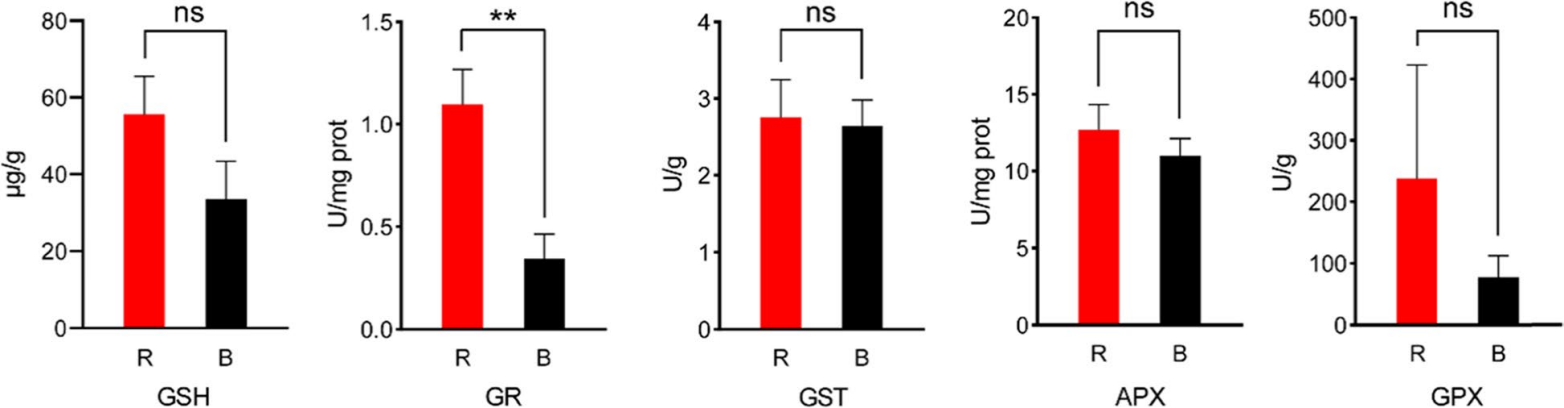
Changes in the enzyme activities of GSH, GR, GPX, APX and GST of maize seedling leaf in response to blue and red-light

GSH being the major anti-oxidative enzyme protects plant from inappropriate light condition. To study the role of blue light and red light on the change of antioxidative properties. The activities of GSH, GR, GPX, APX and GST were determined in maize seedling leaf (Fig. 1). Reduced factor GR decreased the activity of GSH and maintain decreased activity level. The enzyme activity of GR of leaf treated with blue light was significantly lower than that treated with red light. But GSH showed no significant difference in T test. GPX work together with GR to balance the GSSG and GSH. But the activity of GPX was not significant change in response to blue light. The activity of GST and APX involve APX-GSH cycle, no significant different activity reveal that it may be not the main factor affecting GSH metabolism. These experimental outcomes proved that the blue light may induce protective effect in seedling leaf of maize. Activity of enzyme associated with glutathione metabolism primary differ in the GSSG-GSH cycle. Next, we would test this difference by the method of transcriptome and metabonomic.

### Differently expressed genes annotated by Kyoto Encyclopedia of Genes and Genomes (KEGG) and Gene Ontology (GO), and identified by National Center for Biotechnology Information (NCBI) and UniProt in KEGG pathway of glutathione metabolism

**Fig. 2:**
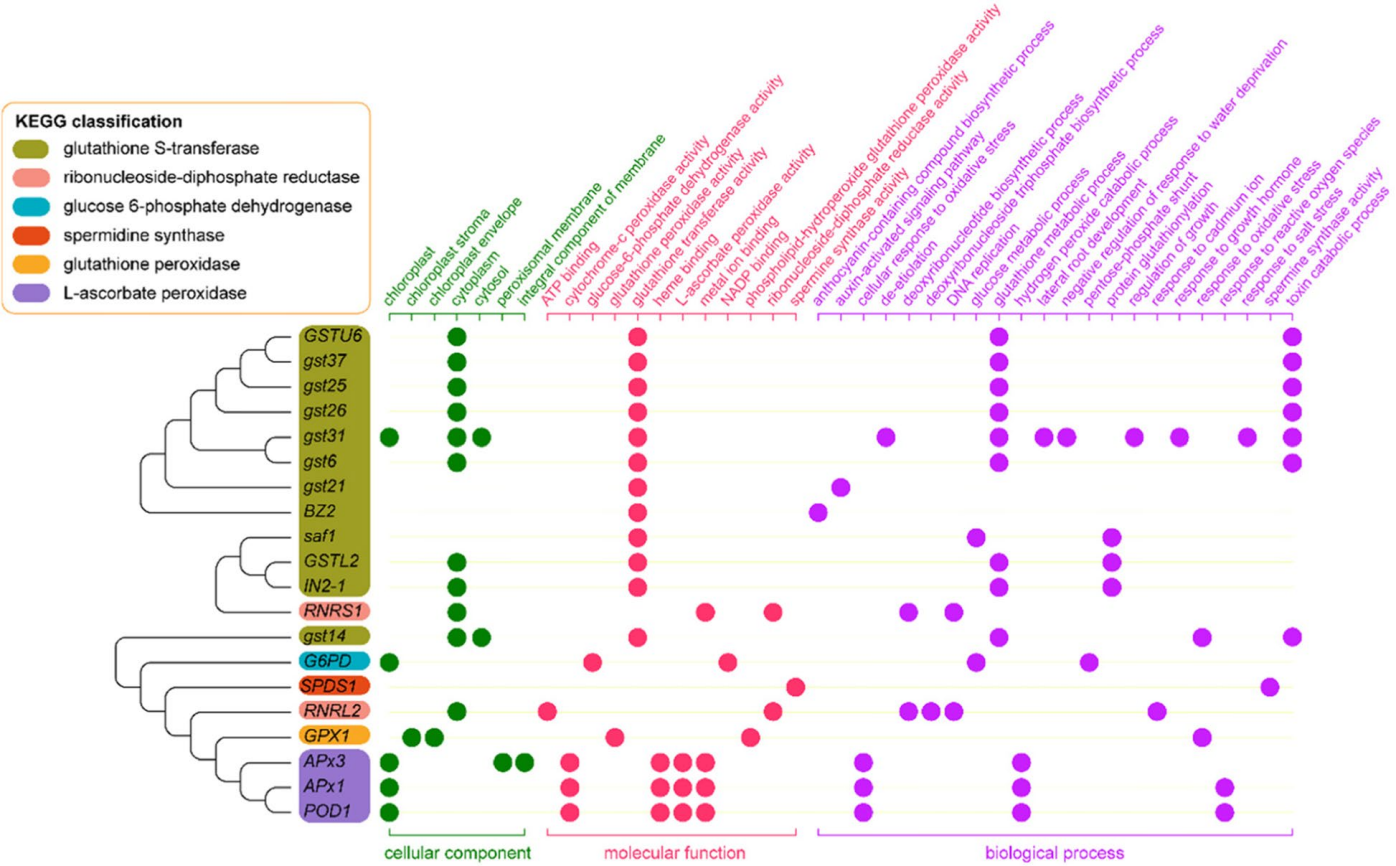
Functional annotations and identifications of 20 DEGs derived from the transcriptome in glutathione metabolism by the KEGG, GO, NCBI, and UniProt of maize seedling leaf

The results of transcriptome sequencing showed that 20 differently expressed genes (DEGs) involved in glutathione metabolism in response to blue light and red light. KEGG analysis showed these 20 DEGs were significantly enriched in “glutathione metabolism” (zam00480) (Fig. 2) which were divided into 6 groups. One of the twenty DEGs could be annotated by the enzyme term glucose 6-phosphate dehydrogenase (NADP, 1.1.1.49). One of the twenty DEGs could be annotated by glutathione peroxidase (1.11.1.9); 1 of the 20 genes could be annotated by spermidine synthase (2.5.1.16); 12 out of 20 genes could be annotated by the glutathione S-transferase (2.5.1.18); 3 out of 20 genes could be annotated by L-ascorbate peroxidase (1.11.1.11); 2 of the 20 genes could be annotated by ribonucleoside-diphosphate reductase (1.17.4.1).

GO analysis was conducted to analyze the functions of these 20 DEGs. The summary of GO categories was depicted in Fig. 2. According to the GO classification, 7, 12, and 22 terms involved in three categories: “cellular component”, “molecular function”, and “biological process”. Eleven, five, and two DEGs involved in the top 3 terms in “cellular component” under “cytoplasm”, “chloroplast”, and “cytosol”. Twelve, four, and three DEGs involved in the top 3 terms in “molecular function” under “glutathione transferase activity”, “metal ion binding”, and “L-ascorbate peroxidase activity”. Nine, seven, and three DEGs involved in the top 3 terms in “biological process” under “glutathione metabolic process”, “toxin catabolic process”, and “cellular response to oxidative stress”.

Phylogenetic tree was drawn to determine the neighbor-joining relationship of 20 DEGs (Fig. 2). Complete annotation of identified DEGs were retrieved from NCBI and UniProt. Among them, 12 GSTs genes belonged to three classes. *gstu6*, *gst37*, *gst25*, *gst26*, *gst31*, *gst6*, *gst21*, and *BZ2* belonged to GSTU (Tau) class. *GSTL2*, *IN2-1*, and *saf1* belonged to GSTL (Lambda) class. *GST14* belonged to GSTF (Phi) class. *RNRS1*, and *RNRL2* were genes of ribonucleoside-diphosphate (rNDP) reductase family. *G6PD* was a gene of glucose-6-phosphate dehydrogenase family. *SPDS1* was a gene of spermine synthase family. *GPX1* was a gene of glutathione peroxidase family. *APx3*, *APx1*, and *POD1* were genes of cytosolic ascorbate peroxidase family. Covariation enrichment of genes sharing the same GO annotation or KEGG pathway annotation.

### Transcriptomics and metabolomics network based on KEGG in glutathione metabolism

**Fig. 3:**
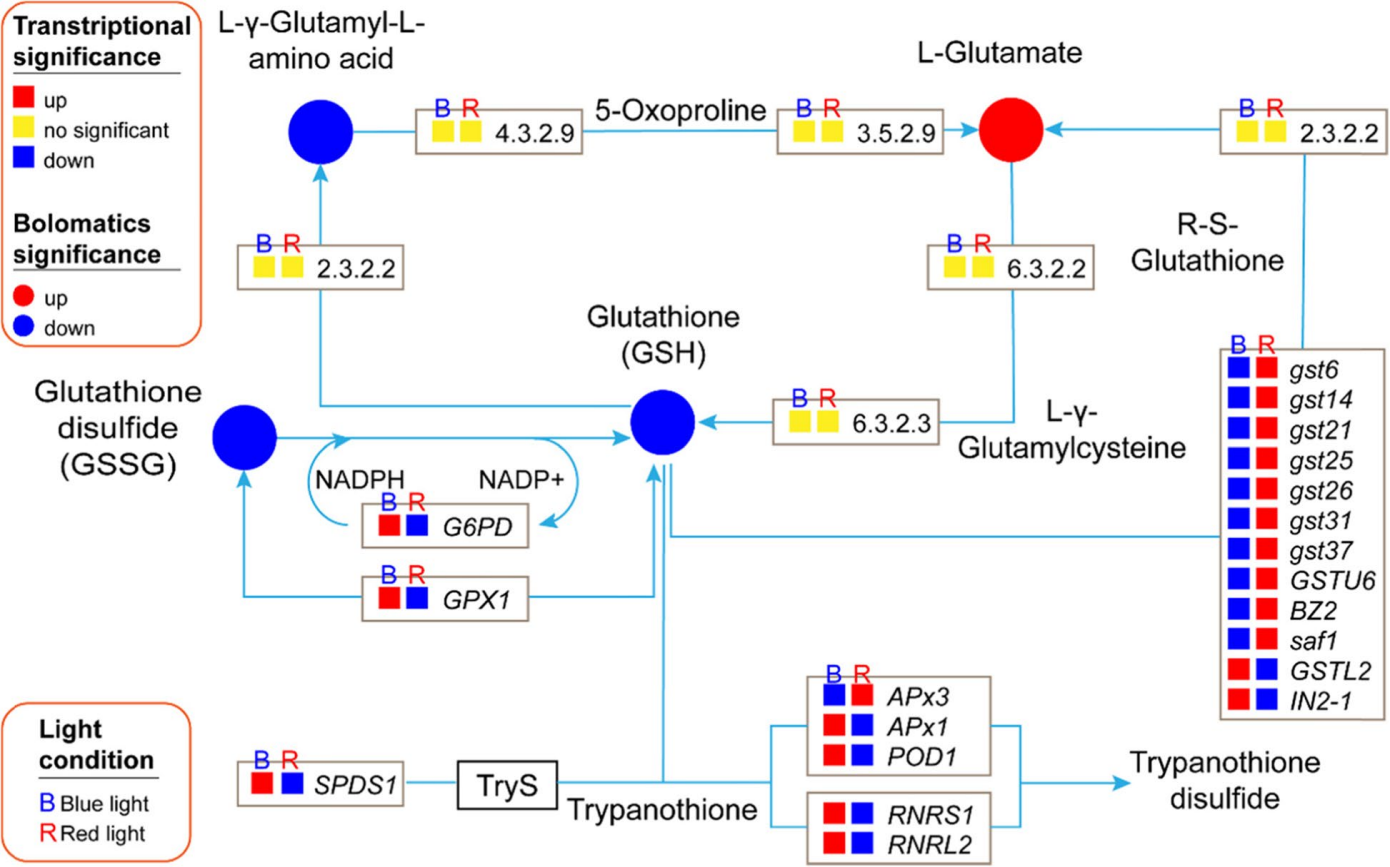
Transcriptional and metabolic regulation network, and significance and position of differentially expressed genes and metabolites in response to blue and red-light based on functionally-enriched KEGG pathways which retrieved from KEGG database in glutathione metabolism

To investigate further, we performed the analysis of transcriptomics profile. In KEGG analysis, 20 DEGs were enriched in glutathione metabolic pathway, among them, 9 DEGs were up-regulated and 11 DEGs were down-regulated in blue light compared to red light (Fig. 3). *G6PD* (1.1.1.49), *GPX1* (1.11.1.9), *SPDS1* (2.5.1.16), *APx1*, *POD1* (1.11.1.11), *RNRS1*, and *RNRL2* (1.17.4.1) were up-regulated. Two DEGs of GSTL class of GSTs family, *GSTL2*, and *IN2-1* (2.5.1.18), were up-regulated. Other 10 DEGs from GSTs family members, *gst6*, *gst14*, *gst21*, *gst25*, *gst26*, *gst31*, *gst37*, *GSTU6*, *BZ2*, and *saf1* (2.5.1.18), were down-regulated. *APx3* of ascorbate peroxidase (1.11.1.11) was down-regulated. There were no significant differences in the transcriptional expression for other Enzyme Nomenclature (EC) of KEGG enzyme (Fig. 3).

The function and interaction of metabolites were constructed using the KEGG database. The enrichment analysis of differently expressed metabolites (DEMs) by analyzing their related metabolic pathways is helpful to understand the mechanism of metabolic pathway changes in different samples. The analysis of the DEMs in glutathione metabolism showed that 4 metabolites accumulated differentially under blue and red-light condition (Fig. 3). Among them, L-glutamate is increased and three metabolic, glutathione (GSH), L-γ-Glutamyl-L-amino acid and glutathione disulfide (GSSG), are decreased. The results of both transcriptome and metabolome analysis indicated that blue and red light affected glutathione metabolism mainly in GSSG-GSH cycle, synthesis L-Glutamate to GSH and APX-GSH cycle. Down-regulation of *G6PD* and *GPX1* in transcriptional level altered the balance of GSSG and GSH. DEGs of GST family affected L-glutamate expression which was a precursor of GSH [17]. *SPDS1*, *APXs* and *rNDPs* interfered the AsA-GSH cycle. These results indicated that transcription expression changed the accumulation of metabolites in glutathione metabolism.

### Identification of potential functional DEGs in glutathione metabolism

**Fig. 4:**
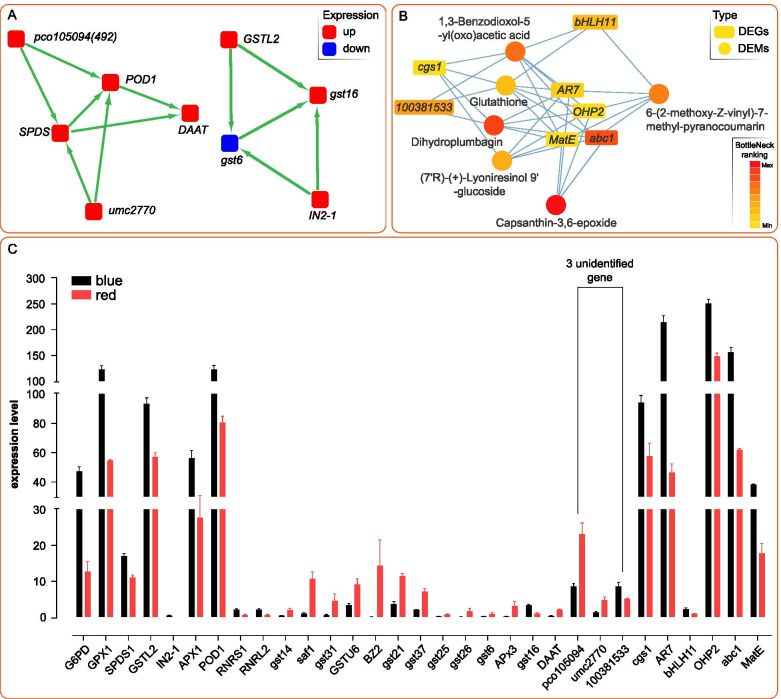
**A** Gene interaction network analysis by 20 DEGs in glutathione metabolism which PPI with all of DEGs in RNA-seq result, consequently, 2 networks constructed by 9 DEGs are obtained. Five DEGs were classified as Cluster 1 and 4 genes were classified as Cluster 2 by MCODE app in Cytoscape software. Red dot represents the up-regulation, blue dot represents the down-regulation. **B** Correlation analysis of DEGs and DEMs revealed that, 7 DEGs and 6 DEMs were correlated in glutathione metabolism in the top BottleNeck ranking analysis method of Cytoscape software. The rectangle node represents the DEGs, the circular node represents the DEMs. **C** RNA-seq expression profiles of 31 DEGs referring KEGG pathway and interaction network of DEGs and DEMs in response to blue and red-light of maize seedling leaf in glutathione metabolism

To understand the gene interaction within glutathione metabolism, we constructed the gene network using the MCODE app of Cytoscape software. The correlation network of 20 DEGs were conducted in all transcriptome samples to fully understand the expression of genes responding to blue and red-light conditions. Nine DEGs, classified into 2 clusters, were obtained. Two clusters I and II contained 5 and 4 genes, respectively (Fig. 4A). In cluster I, two unannotated genes, *pco105094* and *umc2770* were obtained. Both *pco105094* and *umc2770* interacted with *SPDS1* and *POD1* in gene correlation. Meanwhile, two annotated genes, namely *DAAT* and *gst16*, were obtained in cluster I and II, respectively. *DAAT* interacted with *SPDS1* and *POD1*, while, *gst16* interact with *GSTL2*, *IN2-1*, and *gst6* in gene correlation. Combined analysis of transcriptome and metabolome was used to explore potential genes related to glutathione metabolic process (Fig. 4B). Analysis result showed that the co-expression network of DEGs and DEMs consist of 7 DEGs and 6 DEMs. A potential gene *100381533* was detected which interacting with three metabolic, namely, 1,3-Benzodioxol-5-yl(oxo)acetic acid, dihydroplumbagin and glutathione. It should be noted that *abc1* is not annotated in the KEGG pathway of glutathione metabolism, but it is strongly co-related to glutathione metabolism. Additionally, four DEGs, *AR7*, *OHP2*, *MatE* and *abc1* corelated 6-(2-methoxy-Z-vinyl)-7-methyl-pyranocoumarin, (7’R)-(+)-Lyoniresinol 9′-glucoside, and capsanthin-3,6-epoxide with glutathione. *bHLH11* linked with 6-(2-methoxy-Z-vinyl)-7-methyl-pyranocoumarin and Glutathione, *cgs1* linked with (7’R)-(+)-Lyoniresinol 9′-glucoside and glutathione in the co-expression network of DEGs and DEMs.

A total of 31 DEGs were obtained in enriched KEGG pathway, PPI analysis of DEGs in gene correlation network and combined analysis of DEMs and DEGs. Transcriptome profiling indicated that the average relative expression of 16 DEGs were higher than 10, the relative expression of the 5 DEGs were higher than 100. Highly expressed DEGs acted on the GSSG-GSH cycle and AsA-GSH cycle in glutathione metabolism (Fig. 4C).

### Protein sequence and structure analysis of three unidentified genes

**Fig. 5:**
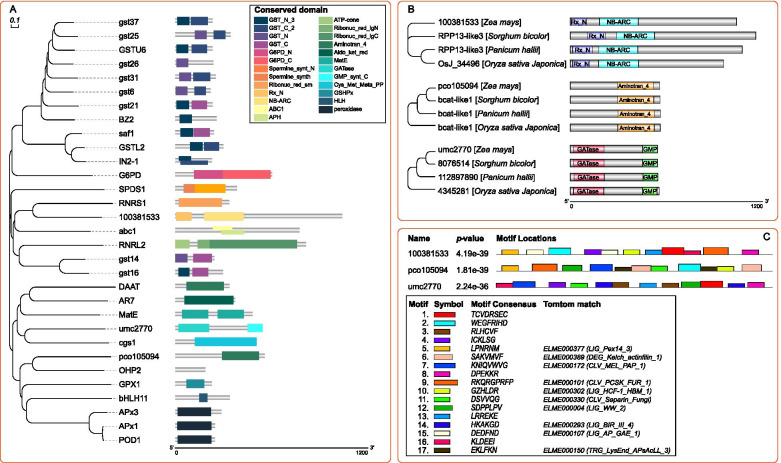
**A** is the phylogenetic tree and conserved domains of protein sequence of 31 DEGs in glutathione metabolism. **B** is the conserved domain of 3 uncharacterized protein 100381533, pco105094, and umc2770 which were blast with 3 related species of *Sorghum bicolor*, *Panicum hallii* and *Oryza sativa Japonica*. **C** is the description of protein motifs of 3 unidentified genes of *100381533*, *pco105094* and *umc2770*

To further explore the evolutionary relationship between unidentified DEGs and identified DEGs. Multiple sequence alignment and phylogenetic tree construction were carried out to determine the neighbor-joining relationship of 31 DEGs (Fig. 5A). After mRNA translated into proteins, the predicted protein domains of 31 DEGs were used to indicate the difference between DEGs. Phylogenetic relationship revealed that 100381533 was closed to RNRS1, umc2770 was a neighbor of cgs1, pco105094 shared a branch with OHP2. In order to further identify the domains of 3 unidentified DEGs. Protein Basic Local Alignment Search Tool (BLASTP) analysis was conducted to predict the conserved domain of 3 unidentified genes by comparison with three Gramineae plants, namely, *Oryza sativa japonica*, *Panicum hallii*, and *Sorghum bicolor* (Fig. 5B). 100381533 was identified as RPP13-like3 which had two conserved domains, Rx_N and NB_ARC. Among three closest related Gramineae plants, the conserved domains of three proteins were similar but their protein were unidentified. Pco105094 had a conserved domain “aminotran_4” which was a domain of “bcat-like 1” protein in other Gramineae plants, so Pco105094 was predicted to be “branched chain aminotransferase proteins like 1” (BCAT-like1). umc2770 was identified as “nucleotide guanosine 5’-monophosphate synthase” (GMPS) with two conserved domains, “GATase” and “GMP-synt_C”. Then, MEME-ChIP tool was used to perform comprehensive motif analysis on 3 unidentified protein associated 3 unidentified genes. A total of 17 motifs were discovered, and 10 of them were similar to known binding motifs, 7 of them were mismatched in Tomtom with any Eukaryotic linear motif resource (Fig. 5C).

**Fig. 6:**
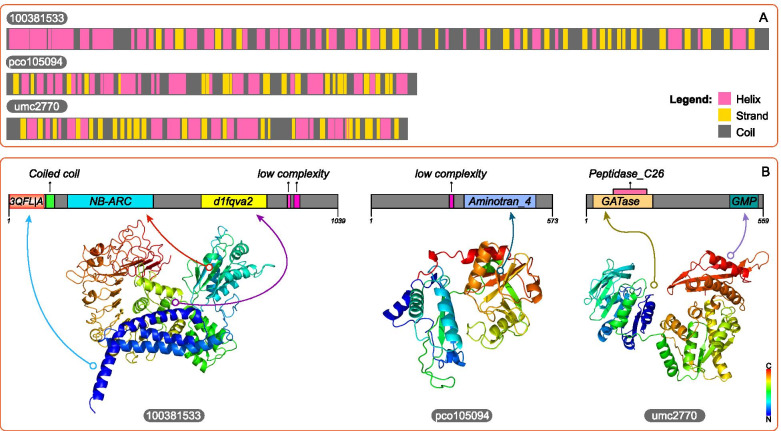
Prediction of secondary (**A**) and tertiary (**B**) protein structure of 3 unidentified genes of *100381533*, *pco105094* and *umc2770*

Protein structure was directly linked to biological functions. After gene sequences translated into amino acids, the amino acids dehydrated and condensed to form peptide chains, and then the peptide chains fold to form tertiary structures to play a role. In order to further define the structure of 3 predicted genes, protein sequence analysis was used to identify the secondary and tertiary structure. The predicted results indicated that pco105904 consisted of 559 aa. Secondary structure was composed of 84 strands, 199 helixes, and 276 coils (Fig. 6A). Tertiary structure model of pco105094 was based on template “c3wwjE”, which followed the PDB title, “Crystal structure of an engineered sitagliptin-producing transaminase, 2 ata-117-rd11”. Two hundred ninety-nine residues (53% of sequence) had been modeled with a single highest score template with 100% confidence. Umc2770 consisted of 545 aa. Secondary structure included 114 strands, 182 helixes, and 249 coils. Tertiary structure model was based on template “c1gpmD”, which followed the PDB title, “*Escherichia coli* GMP synthetase complexed with amp and pyrophosphate”. Four hundred ninety residues (90% of sequence) had been modeled with a single highest scoring template with a confidence of 100% (Fig. 6B). SMART tool was used for further define protein structure. A SCOP domain “d1fqva2” which annotated as “Cyclin A/CDK2-associated p19, Skp2 Human (*Homo sapiens*)”, a coiled coil sequence fragment and 2 low complexity sequence fragments were found in 100381533. A low complexity sequence fragment was found in pco105094. The corresponding positions of the conserved domain and 3D structure were shown in Fig. 6B.

### Predicted position of 3 unidentified genes, and qPCR expression of linking node genes in the network of glutathione metabolism

**Fig. 7:**
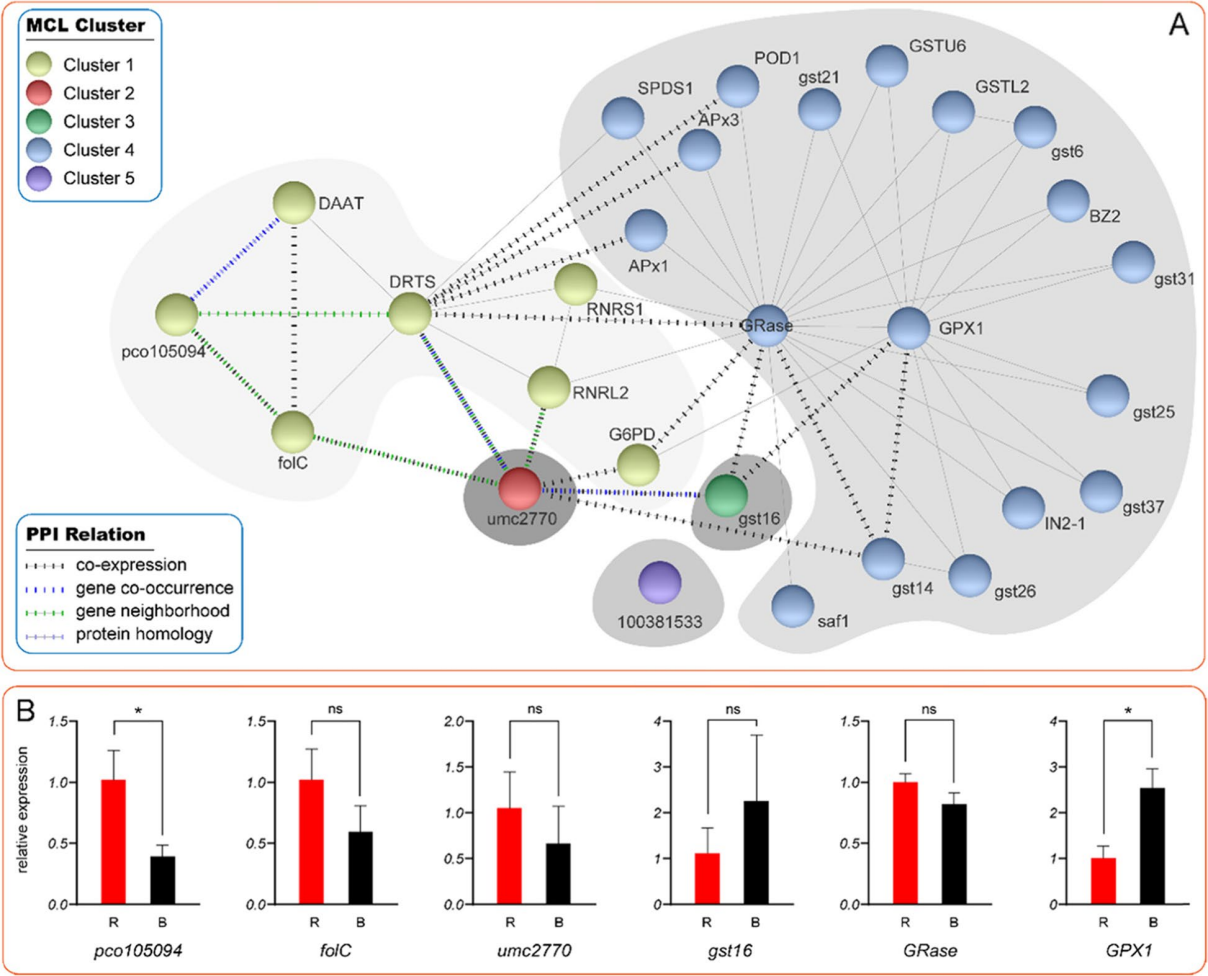
**A** PPI network of 28 proteins of glutathione metabolism in response to blue and red-light was retrieved from STRING 11.0, and was classified into five clusters by MCL method in inflation parameter 10. **B** show the qPCR relative expression of six node genes in the network of glutathione metabolism of maize leaf in response to blue and red-light

To gain further insight into the function of three unidentified genes in glutathione metabolism network, the extended PPI network composed of 28 protein was depicted by String online tool (Fig. 7A). In order to obtain accurate clustering, inflation parameter 10 was used to analyze MCL clustering. Finally, 5 clusters were obtained and two identified proteins of bifunctional dihydrofolate reductase-thymidylate (DRTS) and dihydrofolate synthase (folC) joint the network. In cluster 1, pco105409 co-expressed with folC and had a co-occurrence with DAAT. folC co-expressed with pco105094 and DAAT. In cluster 4, 13 proteins interacted with key node GPX1. gst14 and gst16 were co-expressed with GPX1. GRase was another key node in cluster 4, which played a role of bridge between clusters. umc2770 and gst16 bridged with GPX1, umc2770 and GRase also played as connection of GST protein family with RNRL2, and RNRS1. 100381533 had no protein interaction in the existing maize database. To examine the accuracy of transcript-level expression profile, the gene-level profile of 6 DEGs which linking *pco105094* and *umc2770* with qRT-PCR were compared to those of DEGs with RNA-seq. The relative expression results of 6 genes, *pco105094*, *folC*, *umc2770*, *gst16*, *GRase* and *GPX1* were shown in the Fig. 7B. Compared with red light treatment, the expression of two genes, *pco105094* and *GPX1*, in maize leaf after blue light treatment increased significantly. Although there was no significant difference in qPCR results of other 4 genes, their expression pattern showed concordance in the data.

## Discussion

Glutathione metabolism contains many enzymes and their subsystem metabolic pathways. The differential expressed metabolites (DEMs) and differentially expressed genes (DEGs) enriched by KEGG are concentrated in GSH, APX, GR, GSSH, GST in response to different light conditions. Due to the metabolic cycle and chemical activity of GSH reacting with other compounds, the activity of many compounds in each subsystem metabolic pathways and the level of gene transcription will ultimately determine the activity of GSH and the antioxidant capacity of plants.

### Differently expressed GR and GPX induced by blue light which regulated GSSG-GSH cycle in glutathione metabolism

Glutathione reductase (GR) is a key NADPH-dependent flavo-protein oxidoreductase which can catalyze the glutathione disulfide (GSSG) to reduced glutathione (GSH) and protect plant cells from oxidative damage induced by reactive oxygen species (ROS) burst [18]. GR was co-expressed with G6PD and GPX at protein level, which indicated that glutamine peroxidase and GSSH acted synergistically in glutamine synthesis pathway. However, blue light did not promote the transcription signal and the synthesis of metabolites of these two pathways. In this paper, we obtained one of unidentified genes, pco105094. After transcription for protein, pco105094 was predicted to be an aminotransferase of class IV family protein in UniProt database, namely, branched chain aminotransferase proteins like 1 (BCAT-like1). Two of BCAT proteins were reported to provide the substrates for GSH [19]. BCAT-like1 is a new member, but whether pco105094 is a novel substrate of GSH, which involved in the dominance mediation of GR and G6PD in signaling cascade of GSSG-GSH cycle, still need further experimental evidence. The level of transcription and protease of GPX will affect the conversion of GSH to GSSG [20]. In this experiment, expression of gene-level and transcript-level were both decreased, but enzyme activity of GPX represent no significant change. There may be two reasons for the difference between transcription and enzyme level. The first cause may be the regulated dominance of GR and G6PD signaling cascade. GSH and GSSG were substrates for biosynthesis and biodegradation for each other. Although blue light caused an increase in the transcription level of GPX, the metabolic levels of GSH and GSSG were significantly reduced in GR and G6PD pathways. The second reason may be that plant adaptability stabilized the working efficiency of GPX, which lead to post-transcriptional regulation, thus reducing the activity of GPX enzyme [21–23].

### APX family genes participate in AsA-GSH cycle of glutathione metabolism under blue light

Ascorbate (AsA) and glutathione (GSH) are the two major important non-enzymatic antioxidants in the AsA-GSH cycle. This plays a crucial role in maintaining ROS equilibrium and helps to avoid ROS toxicity [24]. APXs is the first enzyme to catalyze the reaction of H_2_O_2_ into H_2_O, reduced APX as electronic donor. DHAR uses electrons produced by GSH to reduce dehydroascorbate (DHA) and supplies them to APX. It was found that the mRNA expression of ascorbic acid and glutathione, increased with an increase of ROS content in blue light irradiation [25]. Enzyme activity and gene expression of APX were increased in relation to exogenous glutathione [26]. However, cytosolic H_2_O_2_ generated by APx1 deficiency can trigger the adaptation to oxidative stress [27]. *POD1*, another APXs genes, has the same expression is same as *APx1* induced by abio-stress [28]. Unlike *APx1*, *APx3* expression induced by stress is consistent with GSTs gene family [29]. At present, there are few studies on *APx3* in plants. Although they share the same conserved domain and take the similar positions in protein interaction networks but their expression patterns are different. We speculated that a potential APXs signal cascade involved in AsA-GSH cycle. BCAT-like1 may complete this job by working as a new substrate. umc2770 which was predicted to be GMPS which was a bifunctional protein encoding dihydrofolate reductase (DHFR) and thymidylate synthase (TS). homologous protein directly controls the metabolism of aspartate and production of NADPH [30]. Hence, umc2770 was potentially involved in both GSSG-GSH and AsA-GSH cycle, but it is still need further evidence.

### GSTs work in the network of GSH metabolism in response to blue light

Glutathione S-transferases (GSTs) are one of the most important families of detoxifying enzymes in the glutathione metabolism. They are involved in a variety of intracellular events, such as primary and secondary metabolisms, stress metabolism, herbicide detoxification and plant protection against ozone damage, heavy metals, seedling development. GSTs are enzymes that typically add, or substitute, the non-ribosomally synthesized tripeptide glutathione to an electrophilic center contained within a small molecule acceptor [31]. In this paper, we construct a network containing 13 DEGs of GSTs family, and divide the 13 DEGs into 4 subgroups of GSTs family by transcriptome analysis. The network positions of *gst14*, *gst16 and saf1* are different from other genes. The xenobiotic detoxifier gst14 was the most increased protein in maize when exposure to *A. flavus* stress [32]. However, it was decreased in response to metolachlor stress. And the results of expression depends on the sensitivity of varieties [33]. *gst16*, as a new identified DEG belongs to GSTF class like *gst14*. The attack of cereal aphids substantially stimulated the expression of gst16, GPX1, GS in maize [34]. The other 11 GSTs proteins interact with GR and GPX, worked on the procession of anti-stress. But gst16 is predicted to play a key role in the metabolic process leading to early stage brace root development [35]. gst16 co-expressed with GR, GPX1, as well as the unidentified umc2770. This indicates that it does have a different regulatory potential from other GSTs family members. At present, there is little information about saf1, and even different annotations appear in UniProt and NCBI. Our network results show that saf1 can influence glutathione metabolism through co-expression with GR. However, its function needs to be further proved.

### Co-expression network reveals the potential relationship between glutathione with DEGs and DEMs

In addition to core genes and metabolites of glutathione metabolism KEGG pathway, 6 DEGs (*cgs1*, *100381533*, *bHLH11*, *AR7*, *OHP2*, *MatE, abc1*) and 5 DEMs (1,3-Benzodioxol-5-yl(oxo)acetic acid, dihydroplumbagin, 6-(2-methoxy-Z-vinyl)-7-methyl-pyranocoumarin, (7’R)-(+)-Lyoniresinol 9′-glucoside, and Capsanthin-3,6-epoxide) derived from co-expression network those were potentially involved in glutathione metabolism. Because cgs1 serves as a coenzyme for cystathionine which is the precursor of glutathione [36], it is a clear evidence for the correlation of cgs1 and glutathione metabolism. *abc1* was reported to link to drought or other abiotic stresses [37], and its expression was consistent with the trend of GSH expression. bHLH11 also known as CIB1 which bound to glutathione or GST prompted potential redox regulation [38, 39]. Some study presents that pyranocoumarin derivatives display cytotoxic activities [40]. Encoded GST was initially pro- posed to mediated the transfer of glutathione to glucoside derivatives [41]. These studies have partly demonstrated the relationship in our co-expression network. But 4 DEGs (*100381533*, *AR7*, *OHP2*, *MatE*) and 2 DEMs (1,3-Benzodioxol-5-yl(oxo)acetic acid, dihydroplumbagin) still lack of experimental evidence to clarify the relationship with glutathione metabolism. Moreover, here it should be pointed out that fewer such correlations have been reported for maize.

## Conclusions

Our study compared the effects of blue and red-light on glutathione metabolism in maize seedling leaf. Because of the complexity of signal cascade, the influence of specific light signals on glutathione is unpredictable and variable. Combined analysis of transcription and metabolism shows that blue and red-light affect the glutathione metabolism through three transcription signaling pathways. Through the deep excavation of signal network, there are 22 differentially expressed genes in glutathione metabolism. Sixteen of them affected the cycle of glutathione, consequently, changes three metabolites, Glutathione disulfide (GSSG), L-Glutamyl-L-amino acid, and L-Glutamate, significantly different. Finally, it affects the expression of metabolite in glutathione metabolism. Another 6 genes work in APX-GSH cycle, which determines the antioxidant activity of glutathione signaling pathway, are differentially expressed at transcription level. In addition, we mapped a part of signal network through transcription and metabolic analyses, and obtained 3 unidentified genes and correlated metabolites corresponding to signal transduction. The identification of these genes and networks will be helpful to further elucidate the research on the glutathione metabolism of maize.

## Materials and methods

### Plant and light conditions

Xianyu335 as a plant material used in this study were obtained commercially. The formal identification of the plant materials was undertaken by Tieling pioneer seed research company (Tieling, China). The plants are kept at the College of Agriculture, Fujian Agricultural and Forestry University, Fuzhou, China. The maternal part of Xianyu335 is PH6WC and the paternal part is PH4CV. Maize seedlings were cultivated in a climate-controlled cultivation chamber, designed and manufactured by the Center of Excellence for Research in Optoelectronic Agriculture at Fujian Agriculture and Forestry University. Uniform blue and red Light-emitting diode (LED) lamps were distributed on the top of each chamber. Maize seeds were cultivated under two light treatments: 660 nm red (R) and 450 nm blue (B). Light density was set at 150 μmol m^− 2^ s^− 1^. Forty-eight seedlings were cultivated in a light chamber for a 24-h photoperiod every day, and each treatment was repeated four times. The temperature and relative humidity of the cultivation environment were set at 25 °C and 70%, respectively. Each fourth seedling leaf was collected for subsequent experiments.

### Determination of enzyme activity of GSH, GR, APX, GST, and GPX

GST, GPX, GSH, GR, APX were measured using GST Assay Kit, GPX Assay Kit, GSH Assay Kit, GR Assay Kit, and APX Assay Kit, respectively, according to the manufacturer’s instructions from the Beijing Solarbio Science & Technology (Beijing, China). GST assay used 1–chlopro–2,4–dinitrobenzene (CDNB) as a substrate, its activity was determined by monitoring the increase in the absorbance at 340 nm due to GSH–CDNB conjugate accumulation. The amount of GST that catalyze the conjugation of 1 μmol/L GSH with CDNB per minute per mg protein was regarded as one unit. The activity of GPX was determined by catalyzing 1 nmol GSH per minute per mg in samples. The activity of GR was determined by catalyzing oxidation of 1 μmol NADPH per minute per mg in pH 8.0. The activity of APX was determined by catalyzing oxidation of 1 μmol AsA per minute per mg protein. GSH contents was determined by a UV2450 UV/Vis spectrophotometer following the manufacturer’s instructions. Five independent replicates were performed for each treatment.

### RNA sequencing and transcriptome analysis

Total RNA was extracted using the mirVana miRNA Isolation Kit (Ambion) following the manufacturer’s protocol. RNA integrity was evaluated using the Agilent 2100 Bioanalyzer (Agilent Technologies, Santa Clara, CA, USA). The samples with RNA Integrity Number (RIN) ≥ 7 were subjected to the subsequent analysis. The libraries were constructed using TruSeq Stranded mRNA LTSample Prep Kit (Illumina, San Diego, CA, USA) according to the manufacturer’s instructions. Then these libraries were sequenced on the Illumina sequencing platform HiSeqTM 2500 and 125 bp/150 bp paired-end reads were generated. Raw reads of sequencing were processed into clean reads by filtering low quality reads. Then clean reads were mapped to the reference genome of maize B73_RefGen_v4. DESeq was used to analyze differential expression genes (DEGs). *P* value < 0.05 and foldChange > 2 or foldChange < 0.5 was set as the threshold for significantly differential expression. The DEGs were blastp to the related species to establish a predicted protein interaction network. The Cytoscape v 3.5.1 app MCODE was used to develop the considerable modules in the network [42]. Advanced options were 2° cutoff, 0.2 Node Score Cutoff, and 5 K-Core.3.

### Metabolites extraction and LC-MS analysis

Eighty milligrams accurately weighed sample was transferred to a 1.5 mL Eppendorf tube. Two small steel balls were added to the tube. Twenty microliters of 2-chloro-l-phenylalanine (0.3 mg/mL) dissolved in methanol as internal standard and 1 mL mixture of methanol and water (7/3, vol/vol) were added to each sample, samples were placed at − 80 °C for 2 min. Frozen samples were grinded at 60 Hz for 2 min, and ultrasonicated at ambient temperature for 30 min after vortexed, then placed at 4 °C for 10 min. Next, the samples were centrifuged at 13000 rpm, 4 °C for 15 min. Three hundred microliters of supernatant in a brown and glass vial was dried in a freeze concentration centrifugal dryer. Four hundred microliters mixture of methanol and water (1/4, vol/vol) were added to each sample, samples vortexed for 30 s, then placed at 4 °C for 2 min. Samples were centrifuged at 13000 rpm, 4 °C for 5 min. The supernatants (150 μL) from each tube were collected using crystal syringes, filtered through 0.22 μm microfilters and transferred to LC vials. The vials were stored at − 80 °C until LC-MS analysis. QC samples were prepared by mixing aliquots of the all samples to be a pooled sample.

An AB ExionLC UHPLC system (AB SCIEX, Framingham, MA) coupled with an AB Triple TOF 6600 System (AB SCIEX, Framingham, MA) was used to analyze the metabolic profiling in both ESI positive and ESI negative ion modes. An ACQUITY UPLC HSS T3 column (1.8 μm, 2.1 × 100 mm) were employed in both positive and negative modes. The binary gradient elution system consisted of (A) water (containing 0.1% formic acid, v/v) and (B) acetonitrile (containing 0.1% formic acid, v/v) and separation was achieved using the following gradient: 0 min, 5% B; 2 min, 20% B; 4 min, 25% B; 9 min, 60% B; 14 min, 100% B; 16 min, 100% B; 16.1 min, 5% B and 18.1 min, 5% B. The flow rate was 0.4 mL/min and column temperature was 45 °C. All the samples were kept at 4 °C during the analysis. The injection volume was 5 μL. Data acquisition was performed in full scan mode (m/z ranges from 70 to 1000) combined with IDA mode. Parameters of mass spectrometry were as follows: Ion source temperature, 550 °C (+) and 550 °C (−); ion spray voltage, 5500 V (+) and 4500 V (−); curtain gas of 35 PSI; declustering potential, 80 V (+) and − 80 V (−); collision energy, 10 eV (+) and − 10 eV (−); and interface heater temperature, 550 °C (+) and 550 °C (−). For IDA analysis, range of m/z was set as 25–1000, the collision energy was 30 eV.

### Data analysis and bioinformatics analysis

Pearson correlation coefficients were calculated for DEGs and DEMs integration by the method of Cho et al. [43]. For this, the fold changes were calculated in both the metabolome and transcriptome data. And the coefficients were calculated from log2 (fold change) of each metabolite and log2 (fold change) of each transcript using the EXCEL program. Correlations corresponding to a coefficient with R2 > 0.9 were selected. Glutathione metabolism corelated with other DEGs and DEMs were analysis by the cytoHubba app and rank follow BottleNeck ranking method of Cytoscape (3.5.1). Top 12 plots referring glutathione (12 + 1) were selected to drawn the correlation map. Simply id of DEMs was retrieved from ChemSpider (http://www.chemspider.com/). Metabolome and transcriptome relationships were visualized using Cytoscape (version 3.5.1).

R toolkit is used to draw the pictures of transcriptome analysis and metabonomic analysis. The analysis of phylogenetic tree was carried out by MEGA X [44], and depicted by EvolView online tools [45]. Signal network were depicted by Adobe Illustrator CC 2019 (Adobe Systems Incorporated, San Jose, CA, USA). Interaction networks of genes, proteins and metabolites were drawn by Cytoscape 3.5.1 [42] with the method of Liu [46]. Conserved domain and motif of protein were analyzed by Pfam, MEME-CHIP, SMART and SCOP online tools [47–50]. Visualized by DOG2.0 [51]. Protein secondary structure of unidentified protein referring unidentified genes are predicted by online tools of PSIPRED 4.0 (http://bioinf.cs.ucl.ac.uk/psipred/). Phyre2 was used to identify tertiary structural templates of unidentified protein from the PDB (http://www.sbg.bio.ic.ac.uk/phyre2). T test was used to analyze the significant differences between the measured data by comparing their means. The significance level was set at 0.05 (α). Excel 2016 (Microsoft Corporation, Redmond, Washington, USA) was used for multiple comparisons to determine the least significant difference at 0.05 (α).

### qPCR verification

Six genes were selected to perform qPCR. Total RNA was isolated using the RNAqueous® Total RNA Isolation Kit AM1912 (Life Technologies Corp., Grand Island, New York, USA). The RNA yield was determined using a NanoDrop 2000 Spectrophotometer (Thermo Scientific, Waltham, USA), and the integrity was evaluated using agarose gel electrophoresis with ethidium bromide stain. Quantified reactions were performed in a GeneAmp® PCR System 9700 (Applied Biosystems, Waltham, USA). RT-PCR was performed using LightCycler® 480 II Real-time PCR Instrument (Roche, Basel, Switzerland) with QuantiFast® SYBR® Green qPCR Master Mix (Qiagen, Düsseldorf, Germany). Each sample was prepared in three copies for analysis. The primer sequences were designed in the laboratory and synthesized by Generay Biotech (Generay, Shanghai, China) based on the mRNA sequences obtained from the NCBI database. *ZmGAPDH* was used as a housekeeping gene. The sequence for primers used in qPCR were listed in Table S1. Genes were annotated by Nr, Nt, Pfam, KOG/COG, Swiss-Prot, KO, and GO databases, and then applied into GO enrichment analysis and KEGG pathway analysis.

## Supplementary Information

**Additional file 1 **: **Table S1**. The primer sequences of target genes and reference gene in qPCR. 

## Data Availability

The datasets used and/or analysed during the current study are available from the corresponding author on reasonable request. Or these data can be accessed in NCBI SRA database (https://www.ncbi.nlm.nih.gov/Traces/study/?) by PRJNA743131.

## Abbreviations

AsA: Ascorbate
APX: Ascorbate peroxidase
BCAT-like1: Branched chain aminotransferase proteins like 1
BLASTP: Protein Basic Local Alignment Search Tool
CDNB: 1–chlopro–2,4–dinitrobenzene
DEGs: Differently expressed genes
DEMs: Differently expressed metabolites
DHAR: Dehydroascorbate reductase
DRTS: Bifunctional dihydrofolate reductase-thymidylate
EC: Enzyme Nomenclature
folC: Dihydrofolate synthase
G6PD: Glucose 6-phosphate dehydrogenase
GMPS: Nucleotide guanosine 5′-monophosphate synthase
GO: Gene Ontology
GPX: Glutathione peroxidase
GR: Glutathione reductase
GSH: Glutathione
GSSG: Oxidized glutathione
GST: Glutathione S-transferase
KEGG: Kyoto Encyclopedia of Genes and Genomes
LED: Light-emitting diode
NCBI: National Center for Biotechnology Information
NADP+: Dependent isocitrate dehydrogenase
NADPH: Nicotinamide ademine dinucleotidephosphate
ROS: Reactive oxygen species
Spe: Spermidine
SpeE: Spermidine synthase
UV-C: Ultraviolet-C
