# Supplementary material for: Comparative transcriptome and metabolome analysis reveal glutathione metabolic network and functional genes underlying blue and red-light mediation in maize seedling leaf
Source: BMC Plant Biol. 2021 Dec 14;21:593. doi: 10.1186/s12870-021-03376-w (PMC8670197; doi:10.1186/s12870-021-03376-w)
Supplement: Supplementary file 1 — Additional file 1 : Table S1. The primer sequences of target genes and reference gene in qPCR. [file 12870_2021_3376_MOESM1_ESM.docx]

| **Gene Symbol** | **Forward primer (5' to 3')** | **Reverse primer (5' to 3')** |
| --- | --- | --- |
| *pco105094* | GCTGAGGATGCCATTATGCTA | GACAGACTAATGCGTCGTTCA |
| *umc2770* | CAGTACAGACAGTTGGAGTTCAG | AGGCGGCTTAGATGTGATGT |
| *folC(loc103650558)* | CTACTGACAGAGGCGAGCAT | TTCTGGAACTCAGCGAAGGA |
| *gst16(loc542490)* | CAGAACAGAGCCTCGGACAA | GCTGGCATACCAACACCATC |
| *gpm745b(GRase)* | TCTCCATTCCACCACTATCTGTAG | CGCTTGGATATGCTGTTCTTCA |
| *GPX1* | TTGGGTGATCTCGTGAAATGG | TTCTGGATGTCCTTCTCAATCTG |
| *ZmGAPDH* | TACTGTGGATGTCTCGGTTGT | CTGCTGTCACCAAGGAAGTC |
